# Health insurance system fragmentation and COVID-19 mortality: Evidence from Peru

**DOI:** 10.1371/journal.pone.0309531

**Published:** 2024-08-27

**Authors:** Misael Anaya-Montes, Hugh Gravelle

**Affiliations:** 1 Ministry of Economics and Finance, Lima, Peru; 2 Centre for Health Economics, University of York, York, United Kingdom; UT Southwestern: The University of Texas Southwestern Medical Center, UNITED STATES OF AMERICA

## Abstract

Peru has a fragmented health insurance system in which most insureds can only access the providers in their insurer’s network. The two largest sub-systems covered about 53% and 30% of the population at the start of the pandemic; however, some individuals have dual insurance and can thereby access both sets of providers. We use data on 24.7 million individuals who belonged to one or both sub-systems to investigate the effect of dual insurance on COVID-19 mortality. We estimate recursive bivariate probit models using the difference in the distance to the nearest hospital in the two insurance sub-systems as Instrumental Variable. The effect of dual insurance was to reduce COVID-19 mortality risk by 0.23% compared with the sample mean risk of 0.54%. This implies that the 133,128 COVID-19 deaths in the sample would have been reduced by 56,418 (95%CI: 34,894, 78,069) if all individuals in the sample had dual insurance.

## 1 Introduction

Since the first case at the end of 2019 the COVID-19 virus has spread around the world and by 30 June 2021 there had been over 180 million cases and 2 million deaths [1]. The first confirmed case of COVID-19 in Peru was diagnosed on 6 March 2020. By 15 March, the Peruvian Government had declared a national health emergency, instituted a nationwide lockdown, and closed all borders and airports [2]. Peru was the first country in Latin America to implement such stringent social restrictions. Despite these measures, by 30 June 2021, the country reported 2,052,452 COVID-19 infections and 191,948 deaths [3]. With an estimated population of 33 million, this resulted in the highest COVID-19 mortality rate globally, attracting significant attention in academic literature [4–6] and the general media [7–9]. Mortality from COVID-19 varies with age, gender, ethnicity, morbidity, income, and occupation [10–14]. It is also plausible that COVID-19 mortality depends on the resourcing and organization of the health care system, including the way in which the insurance system affects the accessibility of care.

Pre-COVID-19 observational studies either find no or positive effects of health insurance on health outcomes [15–17]. For instance, Miller, Johnson and Wherry [17] used a Difference-in-Difference approach with administrative data to show that Medicaid expansion under the Affordable Care Act (ACA) reduced mortality by 34.9% to 63.2% for those aged 55 to 64. Experimental studies also find that increasing insurance coverage improves health [18, 19]. To date the only study of the implications of insurance coverage for COVID-19 outcomes is Chakrabarti, Meyerson [20] which used a regression discontinuity design to compare counties in US states which had or had not expanded Medicaid coverage under the Affordable Care Act. They found that there were no differences in the logarithms of per capita COVID-19 cases and per capita COVID-19 deaths, though COVID-19 related doctor visits were higher in counties in states which had expanded Medicaid.

Rakus and Soni [21] compared changes in health outcomes before and after the start of the COVID-19 pandemic using data from the 2016–2020 Behavioural Risk Factor Surveillance System, a US nationwide representative survey. They found that for poor individuals Medicaid expansion improved some, but not all, self-reported health outcomes and health-affecting behaviours. Oyeka and Wehby [22] utilised the same dataset from 2017–2021, identifying mental health benefits for certain low-income subgroups during the pandemic. Auty and Griffith [23] examined Medicaid expansion during the pandemic with state-level aggregated data from 2013 to 2020, finding no association between Medicaid expansion and reduced drug or opioid overdose deaths. However, none of these studies specifically addressed COVID-19-related outcomes.

Insurance sub-systems with their own networks of providers typically restrict access to these networks to the individuals they insure. Most previous studies of limited provider networks are based on experience in the US where insurers compete and insureds who chose narrower provider networks can face lower premia [24–26]. There is little evidence on the effect on the health of insureds when access to their provider networks is restricted. Gruber and McKnight [25] found no statistically significant effect of choosing a narrow network on health outcomes such as mortality and avoidable hospitalizations, though confidence intervals were very wide. In the National Health Service of England, where hospital care is tax funded, the relaxation of constraints on patient choice of hospital for coronary artery bypass grafts led to a reduction in post-operative mortality due to some patients switching to higher quality providers [27].

Peru has a fragmented health insurance system in which the two largest sub-systems, which together insured about 80% of the population at the onset of the COVID-19 pandemic, only cover care in their separate networks of providers. In this paper we examine whether this fragmentation contributed to COVID-19 mortality.

Peru has two main healthcare insurance sub-systems. Seguro Integral de Salud (SIS) mainly covers people with low income. SIS provides insurance to households–defined as a set of people who live and eat together, regardless of familiarity. It is financed from general taxation and covered 53% of the 33M population on 5 March 2020. Members of SIS are treated without charge in publicly owned tax-financed providers. The second largest insurance sub-system, covering 30% of the population on 5 March 2020, is El Seguro Social de Salud (ESSALUD) which covers workers in formal employment and their legal dependants (partner and non-adult children). It is financed by compulsory contributions (9% of salary) and provides care free at the point of use.

Some individuals are members of *both* SIS and ESSALUD and so entitled to use providers in both networks sub-systems at no charge. ESSALUD insured individuals can access SIS if they belong to a previously classified eligible household. Conversely, SIS beneficiaries can access ESSALUD by declaring new employment or being recognised as partners of ESSALUD-insured individuals. Some individuals entitled to SIS may choose not to claim SIS if they already have ESSALUD coverage as dependants. Moreover, once a household member eligible for SIS gains formal employment, there is little immediate incentive to declare all dependants to ESSALUD. Together it is possible that some individual can gain dual coverage of SIS and ESSALUD simultaneously managing the time of their affiliation for both sub-systems.

Furthermore, while policymakers strive for universal insurance coverage, holding both SIS and ESSALUD simultaneously is viewed as cross subsidising ESSALUD, prompting disaffiliation processes. The Superintendencia Nacional de Salud -SUSALUD centralise all the insurance information nationwide. However, since SUSALUD relies on data from SIS and ESSALUD, its ability to detect dual insurance depends on the frequency of data transfers between these sub-systems. Delays in data transfers can prolong the disaffiliation process for weeks or months.

We use a definition of dual insurance based on insurance status before the start of the pandemic, specifically having dual insurance for at least seven consecutive days between 1 January 2020 and 5 March 2020, this is because using information on insurance status after the start of the pandemic creates the risk of reverse causality, i.e becoming infected with COVID-19 (and hence at risk of death from COVID-19) may make eligible individuals more likely to claim dual insurance if they do not already have it or to contest attempts to remove them from one of the sub-systems if they do.

We use data on 24.74M individuals who were in one or both sub-systems before the start of the pandemic in Peru (6 March 2020). Of these 94.17% were in only one of the sub-systems and 5.83% were in both sub-systems and so could access providers in both networks. We compare COVID-19 mortality risk of those in only one sub-system and those in both sub-systems (dual insurance).

The next section outlines the characteristics of the Peruvian healthcare system and describes the datasets. Section 3 sets out the empirical model, the rationales for using bivariate probit as the preferred estimation method and discusses the instrument variable. Section 4 has the results for the biprobit models of COVID-19 mortality risk estimated on 24.74M individuals. We also compare the biprobit model results with those from models which make different assumptions about functional form and the endogeneity of dual insurance, and different definitions of dual insurance. Section 5 analyses the findings in relation to previous research and considers the policy implications, while Section 6 provides the conclusion.

## 2 Background and data

Since the onset of COVID-19 in Peru, the government has established new datasets at the patient level, which include information on mortality and insurance status. This section discusses the context of health system fragmentation, the mechanisms through which individuals can obtain dual insurance, and the administrative datasets used to analyse dual insurance and its impact on COVID-19 mortality.

### 2.1 Health system fragmentation in Peru

Historically, the development of Peru’s healthcare system has been characterised by fragmentation, especially in terms of financing and healthcare provision. Fig A1 in S2 Appendix illustrates the Peruvian health system, which includes financiers—mainly health insurers—and providers operating within two sub-systems: public and private. This results in a mix of public financiers with public providers and private financiers with private providers.

The first health insurance initiative in Peru commenced in 1924 with the establishment of the Servicio de Salud de la Policía for military and police personnel [28]. Over time, it expanded into four institutions: the Fondo de Aseguramiento en Salud de la Policia (under the Ministry of Interior) and the Fondo de Aseguramiento en Salud for the three branches of the Armed Forces (under the Ministry of Defence), each with its own insurance and healthcare providers. These closed sub-systems cover only their respective members and their families, representing about 1.5% of the population by the pandemic’s start. This highlights the fragmentation within Peru’s health insurance and provision system, even among police and military personel.

In 1936, the Caja Nacional del Seguro Social was established for the working-class population. In 1948, the Caja Nacional del Seguro Social de Empleados was created for employee insurance. These merged in 1973 and became El Seguro Social de Salud (ESSALUD) in 1997 under the Ministry of Labour [29]. ESSALUD, covering 30% of the population as of March 2020, insures workers in formal employment and their dependents, funded by compulsory contributions (9% of salary), and operates its own hospitals which means that only ESSALUD insureds can access them. ESSALUD can only be accessed through the formal labor market. Therefore, workers in the informal labor market are either covered by SIS or remain uninsured.

The Ministry of Health (MINSA), established in 1935, began insuring uninsured people in the late 1990s with the Seguro Escolar Gratuito and Seguro Materno Infantil, merging into the Seguro Integral de Salud (SIS) in 2001. SIS, initially for the impoverished, expanded over time and covered 53% of the population as of March 2020. SIS provides free treatment in publicly funded providers and extended coverage to all uninsured individuals after our analysis period.

Public health facilities, funded by general taxes and administered by regional governments or the Health Ministry in Lima, offer subsidised services. These public providers also treat non-SIS members, including ESSALUD insureds and the 15% of the population who were uninsured at the start of the pandemic, at subsidized prices. SIS buys services from these public facilities but does not own or administer them.

Private insurance, dominated by Entidades Prestadoras de Salud (EPS), covers 8% of the population but only 1.5% exclusively by the start of the pandemic, receiving subsidies from ESSALUD contributions. Private insurance buys services from private health providers, which ofen charge co-payments to the insured. Uninsured invividuals and SIS or ESSALUD insureds can also access private providers paying market prices fees.

In summary, Peru’s insurance system has evolved in a fragmented manner, illustrated by the subdivision of health insurers for police and military personnel. Despite unification efforts, particularly within ESSALUD and SIS, the public health insurance system is currently under four ministries: SIS (Ministry of Health), ESSALUD (Ministry of Labour), Police (Ministry of Interior), and Armed Forces (Ministry of Defense). This fragmentation extends throughout the Peruvian public sector, including the Parliament, where the Ministry of Health is overseen by one parliamentary committee while ESSALUD, linked to the Ministry of Labour, falls under another. This study focuses on the two largest public insurers, SIS and ESSALUD, which together covered more than 80% of the population at the start of the COVID-19 pandemic.

### 2.2 Dual insurance SIS and ESSALUD

Information on individual insurance status is from the SUSALUD-RAUS database [30] which combines data from all insurance sub-systems. It provides a day-by-day history of insurance status for all citizens from their first insurance enrolment in any health insurance sub-system.

Some individuals are members of *both* SIS and ESSALUD and so entitled to use providers in both networks sub-system at no charge. In the following, we discuss the rules of enrolment that can explain why some people can have both insurance at the same time.

Upon employment, all workers are automatically and compulsorily enrolled in ESSALUD insurance through employer declaration to the tax authority, involving a 9% salary deduction. Dependants (partners, children under 18, and adult children with severe disabilities) qualify for coverage but require explicit registration. Until registration, dependants may be covered by SIS if eligible. Job loss terminates ESSALUD coverage, with a grace period based on the number of monthly contributions during the employment period before its end, during which affiliation with SIS can be sought if eligible.

ESSALUD has potential affiliation issues. Employers can create fictitious employment scenarios, legally paying the 9% of the employee’s minimum salary to ESSALUD to secure health coverage for the employee and their dependants. There were cases that ghost businesses were created to provide ESSALUD health insurance to only one employee. Individuals can be declared domestic workers without formal business setup, paying 9% of minimum salary, with no age restrictions, potentially including the very ill or elderly. ESSALUD permits partners to be accredited with a mere sworn declaration, lacking substantial proof. The primary worker must be legally single, divorced, or widowed. In 2015, ESSALUD disaffiliated 5,521 insured individuals due to fraudulent affiliations. These involved employers not declaring other taxes besides ESSALUD contributions, employers with only one employee, multiple partners and concubines, and domestic workers [31].

SIS primarily employs household socioeconomic classification facilitated through the Sistema de Focalización de Hogares (SISFOH). This classification remains valid for 4 years in urban areas, 6 years in rural areas, and 8 years within indigenous Amazonian populations. Eligibility for SIS is contingent upon households being classified as poor or extremely poor. Moreover, SIS extends coverage to specific demographics irrespective of socioeconomic status, including expectant mothers up to 42 days post-childbirth, children under 5 years old, and individuals who were victims of terrorism between 1980 and 2020, among other groups, provided they were uninsured at the time of enrolment.

It should be noted that the employment of a household member eligible for SIS, which results in ESSALUD affiliation, does not automatically update the household’s deprivation classification. Furthermore, households have the option to request revisions in their classification through local governmental bodies to qualify for SIS and other social programmes, although this process may introduce inaccuracies as this is handled by local goverments that can seek to be seen benevolent from their electoral population.

Entitlement to SIS does not equate to automatic enrolment. Before the COVID-19 pandemic, individuals usually had to proactively begin the enrolment process at local health providers, which affiliated individuals to SIS that latter were recorded in the RAUS-SUSALUD database.

In this context, ESSALUD insured individuals can access SIS if they belong to a previously classified eligible household, as socioeconomic classification does not immediately change with formal employment. The reaffiliation process to SIS can be enacted at any time, and due to delays in information sharing, it is possible for individuals to be covered by both SIS and ESSALUD simultaneously.

Conversely, SIS beneficiaries can access ESSALUD by declaring new employment or being recognized as partners of ESSALUD-insured individuals. Some individuals entitled to SIS may choose not to claim it if they already have ESSALUD coverage as dependents, opting to use SIS only when necessary. Moreover, once a household member eligible for SIS gains formal employment, there is little immediate incentive to declare all dependents to ESSALUD. Dependents can remain enrolled in SIS and be declared to ESSALUD as needed, allowing for dual coverage at their discretion.

Furthermore, while policymakers strive for universal insurance coverage, holding both SIS and ESSALUD simultaneously is viewed as a source of financial pressures to SIS, prompting disaffiliation processes. SUSALUD shares with SIS and ESSALUD information of dual insurance cases, typically resulting in disaffiliation from SIS. However, since RAUS-SUSALUD relies on data from SIS and ESSALUD, its ability to detect dual insurance depends on the frequency of data transfers between these systems. Delays in data transfers can prolong the disaffiliation process for weeks or months.

**Fig 1:**
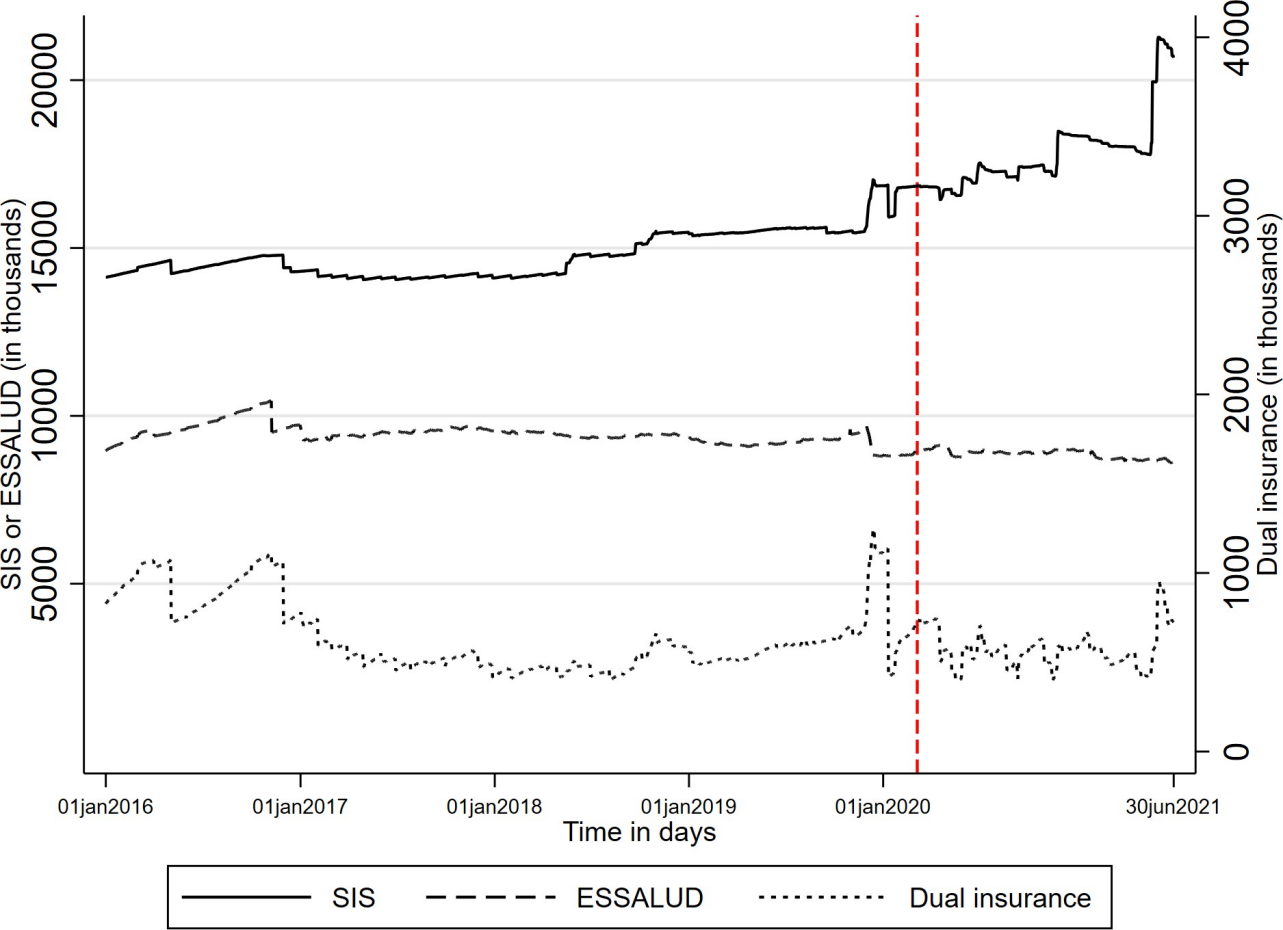
Daily number of insureds by ESSALUD, SIS and dual insurance status 1^st^ January 2016–30 June 2021. Note. Figure plots the daily number in SIS, ESSALUD (left hand scale) and in both insurance sub-systems (right hand scale). The vertical line shows the start of the pandemic.

**Fig 1** illustrates the daily count of individuals insured under SIS, ESSALUD, and both sub-systems from 1 January 2016 to 30 June 2021. In January 2016, approximately 14 million people were insured under SIS, about 9 million under ESSALUD, and just under 1 million held dual insurance under both sub-systems simultaneously. From December 2019, basic SIS coverage was extended to all uninsured individuals, though implementation was partial. The number of SIS-insured individuals showed a slight but steady increase before the pandemic, followed by a significant rise after the onset of the pandemic period, exceeding 21 million by 30 June 2021.

Conversely, the count of ESSALUD-insured individuals remained relatively stable, with a slight decline during the pandemic, indicating a continued process of disaffiliation due to job losses. By 30 June 2021, there were slightly fewer individuals insured under ESSALUD compared to January 2016.

The number of individuals with dual insurance exhibited instability and irregular trends. In the first half of 2016, approximately 100,000 insured individuals were disaffiliated. Despite this, dual insurance remained prevalent, with over 1 million individuals by September 2016. Another significant disaffiliation at the end of 2016 reduced the number of dual-insured individuals to about 500,000, a figure that remained relatively stable, with some fluctuations, throughout 2017 and 2018.

Fig A2 in S2 Appendix show no specific pattern of massive affiliations or disaffiliations for dual insurance from January 2018 to November 2019. However, notable events included nearly 100,000 affiliations to dual insurance on certain days in December 2019 and a mass disaffiliation of approximately 700,000 insured individuals in January 2020.

During the pandemic, the frequency of affiliation and disaffiliation processes increased. This suggests that individuals entitled to dual insurance claimed it more during emergencies like the COVID-19 pandemic, where access to providers could improve survival chances. Meanwhile, the system responded by disaffiliating more individuals. Fig A3 in S2 Appendix illustrates that more people were affiliated and disaffiliated for dual insurance during the pandemic compared to before it began.

Policymakers’ attitudes towards dual insurance appear to change over time. Fig A4 in S2 Appendix shows the same information as Fig 1 but over an extended period, from 1 January 2010 to 30 June 2021, using RAUS-SUSALUD records. RAUS-SUSALUD started collecting information in 2012. Thus, historical information before this period does not reflect the true number of people affiliated with each sub-system as it was collected retrospectively. This also means that before this period, it was not possible to detect dual insurance or remove individuals who claimed it due to a lack of information. From 2012 to 2013, the number of people with dual insurance began to increase and become visible to insurance administrators. However, there was no evidence of disaffiliations of dual insurance. The first massive disaffiliation occurred in January 2014, and since then, recorded affiliations and disaffiliations of dual insurance have become more common.

The tension between extending health insurance coverage to uninsured individuals while curtailing coverage for those with dual insurance is evident in Peru’s normative developments. The Universal Health Insurance Law (Law 29344) [32], enacted in 2009, mandated universal health insurance coverage, which facilitated the expansion of SIS coverage. This expansion was further supported by Emergency Decree 017–2019 in 2019 [33], aimed at providing basic SIS coverage to uninsured individuals, albeit with limited success. It was only with the onset of the COVID-19 pandemic that administrative data tracking COVID-19 mortality rates highlighted significantly higher mortality among the uninsured. A working paper commissioned by the Ministry of Economics of Peru revealed that the case fatality rate for uninsured individuals was approximately ten times higher than for those with insurance during the initial nine months of the pandemic (see Table A1 in S2 appendix). In response, Urgency Decree 046–2021 [34] was enacted, allocating financial resources to ensure that by the bicentenary of the Peruvian Republic in July 2021, all uninsured individuals would be covered by SIS insurance. However, this decree was implemented after the analysis period of this study, which spans from 6 March 2020, the date of the first confirmed COVID-19 case, to 30 June 2021.

Despite efforts to expand insurance coverage, Supreme Decree No. 008-2010-SA from April 2010 [35], which develops the Universal Health Insurance Law, stipulates in Article 78 that affiliation with one insurance sub-system excludes others, thereby preventing dual insurance claims. Initial enforcement of this provision was impeded by the lack of a unified database, as previously noted. In November 2014, Supreme Decree No. 030-2014-SA [36] required SIS to check for dual insurance within RAUS-SUSALUD. Article 30 of this decree recognised potential delays in updating information and allowed SIS to seek financial reimbursement from other sub-systems if individuals covered by SIS claimed health services being affiliated to another health insurance. This implicitly acknowledged the possibility of dual insurance.

The drive to eliminate dual insurance is largely financial, aiming to prevent SIS resources from subsidising ESSALUD, which is funded by employment contributions. The implementation of disaffiliation policies also depends on political will, which has fluctuated over time. Between 2014 and 2021, Peru had five presidents, 13 health ministers, and nine SIS heads, each potentially adopting different stances on dual insurance. To reinforce disaffiliation policies, administrative resolutions were periodically issued by SIS. For instance, Resolución Jefatural 126-2015/SIS in 2015 [37] and Resolución Jefatural No 112-2020/SIS in 2020 [38] both reinforced disaffiliation for individuals with other health insurance.

These policies aimed to prevent SIS from subsidising ESSALUD but overlooked the potential health benefits of dual insurance, such as reduced mortality. Our contribution highlights the health benefits of dual insurance, advocating for its extension and generating evidence to support the unification of all health insurance sub-systems.

### 2.3 COVID-19 mortality

Information on COVID-19 mortality is from the NOTI-SINADEF COVID-19 mortality database [39] which was established in response to the pandemic. The initial mortality classification system used in Peru until 31 May 2021 led to a substantial undercount of COVID-19 deaths. With the initial system the ratio of excess deaths (all deaths minus the number predicted from all-cause mortality in the five previous pre-pandemic years) to reported COVID-19 deaths was 2.7, with a correlation between the weekly figures of 0.87 [5]. Revised classification criteria [40] were introduced in May 2021 and applied retrospectively to mortality from 1 March 2020 to 31 May 2021 (for details see S1 Appendix). This led to the number of deaths attributed to COVID-19 over this period more than doubling, the ratio of excess deaths to COVID-19 deaths decreasing to 1.0, and the weekly correlation between excess deaths and COVID-19 deaths increasing to 1.0 [5]. This suggests that the COVID-19 mortality data based on the revised definition that we use for 6 March 2020 to 30 June 2021 are reasonably accurate.

We use the insurance and mortality databases to construct the sample for analysis of the effect of dual insurance on COVID-19 mortality. In addition to daily information on insurance status (in SIS, in ESSALUD, or in both) and mortality, the databases record gender, age, and district of residence. Districts are the smallest administrative unit. The 1,874 districts have an average population of 17,600 and a radius of 10.8km. They are grouped within 196 provinces, which in turn are grouped in 25 departments. We use the district of residence to attach measures of district poverty, population, area, district geographical type (capital city, coast, jungle, mountain) and the straight line distance from district centroids to hospitals in the SIS and ESSALUD networks.

After dropping individuals with missing data or who were not in SIS or ESSALUD for at least seven consecutive days during 2019 and for seven consecutive days between 1 January 2020 and 5 March 2020, our sample for the analysis of the effect of dual insurance on mortality from COVID-19 is 24,739,933 of whom 0.54% died from COVID-19 between 6 March 2020 and 30 June 2021. Table A2 in S2 Appendix has details of data cleaning.

## 3 Methods

We estimate individual level biprobit models to allow for the small probabilities of dual insurance and of COVID-19 mortality in our sample. It is possible that unobserved variables affect both whether an individual has dual insurance and whether they die from COVID-19. We allow for such possible endogeneity bias by using an instrumental variable (IV): the *difference* in the distance to the nearest provider in each of the two networks. The IV is a measure of the improvement in access to care from being in both insurance sub-systems rather than only one sub-system and so is a good predictor of having dual insurance. We argue that, since we control for individual’s characteristics and the characteristics of their district of residence (including the structural quality of, and distances, to providers in both networks, COVID-19 infection rates), the IV satisfy the exclusion requirement that it is not, conditional on the covariates, correlated with COVID-19 mortality risk.

### 3.1 Endogeneity of dual insurance

Whether an individual dies from COVID-19 and whether they have dual insurance may both be affected by the same unobservable factors, thereby potentially biasing the estimated effect of dual insurance on COVID-19 mortality risk. Less healthy individuals may be at greater risk of death from COVID-19 but be more likely to claim dual insurance if eligible because they believe they are more likely to benefit from having better access to providers when ill. This will lead to an underestimate of the reduction in COVID-19 mortality risk from having dual insurance. Conversely more health-conscious individuals may take better care of their health, thereby reducing their COVID-19 mortality risk, and be more likely to want dual insurance. This will lead to an over-estimate of the beneficial effect of dual insurance. There may also be endogeneity when dual insurance status is affected by decisions by insurance system officials whose interpretation of eligibility rules may depend on their perception of the benefits or cost implications of dual insurance. As discussed in subsection 2.2, although there are rules to prevent individuals from holding dual insurance, officials more concerned with financial impacts may enforce these rules more strictly than those who are aware of potential positive health impacts.

We allow for possible endogeneity bias by using an instrumental variable (IV) which predicts having dual insurance and is not correlated with mortality except via its correlation with dual insurance. The IV is the *difference* in the distance from an individual to the nearest provider in each of the SIS and ESSALUD networks. This differential distance IV draws on a literature dating back to McClellan, McNeil and Newhouse [41], which recognises that distance is a critical factor influencing patients’ utilisation of healthcare services. While the actual distance travelled by patients may be endogenous—since more severely ill patients may travel further to access higher quality care—the fixed geographic distance between a patient’s location and healthcare providers is not influenced by the patient’s health status or provider choice. An expanding body of literature in health economics uses distances as instrumental variables, following McClellan’s methodology. For example, Gowrisankaran and Town [42] employed the distance between patients and hospitals as an IV to assess the quality of pneumonia care in Southern California from 1989 to 1994. Similarly, Kahn, Ten Have and Iwashyna [43] used the distance from a patient’s home to the nearest high-volume hospital as an IV to investigate the relationship between hospital volume and mortality rates in cases requiring mechanical ventilation. Additionally, Berrie, Feng [44] utilised the road distance from home to the nearest cycle path as an IV to study the impact of cycle commuting on mental health.

SIS and ESSALUD have different numbers of insureds, and different numbers of providers with different levels of equipment and facilities. For those eligible for both insurance sub-systems, the gain from having dual insurance rather than single insurance will depend on the *differences* between the SIS and ESSALUD providers they could access as members of only one sub-system. Individual *i* and resident in district *r* who is initially only in SIS but is also eligible for ESSALUD will be more likely to also be in ESSALUD the greater the distance ($d_{ir}^{SIS}$) to the nearest SIS provider compared with the distance $\left(d_{ir}^{ESS}\right)$ to the nearest ESSALUD provider: $\left(d_{ir}^{SIS}-d_{ir}^{ESS}\right)$. Conversely, those initially in ESSALUD but eligible for SIS will be more likely to get dual insurance the greater is $\left(d_{ir}^{ESS}-d_{ir}^{SIS}\right)$. Our distance based IV for dual insurance is

$$z_{ir}=S_{ir}^{first}(d_{ir}^{SIS}-d_{ir}^{ESS})+(1-S_{ir}^{first})(d_{ir}^{ESS}-d_{ir}^{SIS})=(2S_{ir}^{first}-1)(d_{ir}^{SIS}-d_{ir}^{ESS})$$ (1)

where $S_{ir}^{first}$ is an indicator for the individual initially being in SIS. We use individual insurance history from the insurance database SUSALUD-RAUS to determine which sub-system the individual was in first. The larger the value of $z_{ir}$ the greater is the reduction in distance from having dual insurance rather than single insurance and hence the more likely is it that an individual will opt to have dual insurance if they are eligible for both sub-systems.

We measure $d_{ir}^{SIS}$ and $d_{ir}^{ESS}$ as straight line distances, using their personal address for 62% of individuals and the district centroid as the location for the remainder. Using official government classification we also control for the rurality of the district and its geographical characteristics (jungle, mountain, coastal region, capital city) which may be correlated with transport facilities and ease of access.

If an individual infected with COVID-19 seeks hospital treatment for the disease, the distance to the provider they use may affect their mortality risk. Although we do not observe in which, if any, hospital an individual infected with COVID-19 is treated, the covariates in the mortality model include characteristics of local providers (quintiles of the average distance to the nearest SIS and ESSALUD provider, and the average level of providers of the nearest SIS and ESSALUD providers), as well as the proportion of the district population infected with COVID-19 as a measure of the capacity of local providers to treat COVID-19 cases. We argue that, conditional on the individual and district covariates in the mortality model, our IV based on *differences* in distances to the nearest providers in the two networks will not be correlated with the individual’s mortality risk and so satisfies the exclusion requirement.

### 3.2 Definition of dual insurance

Using information on insurance status *after* the start of the pandemic creates the risk of reverse causality. Becoming infected with COVID-19 (and hence at risk of death from COVID-19) may make eligible individuals more likely to claim dual insurance if they do not already have it or request reaffiliation if they have been removed from one of the sub-systems. Fig A5 in S2 Appendix plots dual insurance status for those who died from COVID-19 for 100 days up to the date of death and shows that the number with dual insurance increased in the month before death, suggesting that using dual insurance data from *after* the start of the pandemic risks capturing the pandemic’s impact on dual insurance—introducing reverse causality that should be avoided. Similarly, we refrain from using other information *after* the start of the pandemic, such as hospitalization, consultations, or referrals between providers, as covariates or control variables. These variables could be influenced by patients’ ability to access healthcare services, which might be mediated by their ability to obtain dual insurance, potentially affected by the risk of COVID-19 mortality. Furthermore, some individuals died from COVID-19 before accessing any healthcare provider—possibly while waiting for hospital admission. These deaths were later confirmed to be due to COVID-19 through epidemiological investigation methods detailed in S1 Appendix. We therefore use a definition of dual insurance based on insurance status *before* the start of the pandemic. We argue that having dual insurance for at least seven consecutive days between 1 January 2020 and 5 March 2020 is a good predictor of being able to get dual insurance if the individual is infected with COVID-19. We also consider a range of alternative definitions and obtain similar results.

A narrow definition of dual insurance (for example having dual insurance for *all* days in a particular pre-pandemic period) risks classifying some people who had dual insurance for only part of this period but who were able to get dual insurance during the pandemic period as not having dual insurance. Similarly, defining dual insurance as having dual insurance on a particular day also risks classifying those who had dual insurance on other days and were able to get dual insurance during the pandemic as not having dual insurance. Conversely, a wide definition (for example, having dual insurance on *any* day within a long pre-pandemic period risks classifying some individuals who could not get dual insurance during the pandemic period as having dual insurance. Both types of classification error will lead to an underestimate of the effect of dual insurance on mortality risk. If some of those classified as not having dual insurance pre-pandemic did get it during the pandemic, this will reduce the average mortality risk for those classified as not having dual insurance compared to those classified as having dual insurance. If some of those classified as having dual insurance pre-pandemic did not have it during the pandemic, this will increase the average mortality risk for those classified as having dual insurance compared to those classified as not having it. We examine the sensitivity of estimates of the effect of dual insurance on mortality risk to using a variety of different pre-pandemic definitions of dual insurance.

### 3.3 Model specification

Two stage least squares (2SLS) is simple and provides a linear approximation to the conditional expectation outcome function [45]. When the IV for dual insurance is continuous the 2SLS coefficient on the dual insurance indicator in the mortality model is an average of local average treatment effects (LATEs) for individuals whose dual insurance status is affected by the instrument. 2SLS can yield estimates of the average effect of treatment (ATE) and the average effect of treatment on the treated (AETT) but these require stronger assumptions [46–48]. However, our outcome (mortality) and potentially endogenous treatment (dual insurance) are binary variables with small probabilities and, as we show, this can lead to linear models producing predicted probabilities for individuals outside the [0,1] interval. It is also possible for linear models to produce estimates of average treatment effects (ATE) with the wrong sign [49, 50].

The obvious alternative to 2SLS is the non-linear recursive bivariate probit (biprobit) specification [51, 52]. Biprobit requires stronger assumptions than 2SLS but estimates the average effect of treatment (ATE) and the average effect of treatment on the treated (AETT). A simulation study by Chiburis et al. (2011) suggests that biprobit is more efficient than 2SLS, especially in models with covariates and when, as in our case, the probability of treatment (dual insurance) is low. Other simulations also support the use of biprobit rather than 2SLS [53, 54]. Although Chiburis, Das and Lokshin [55] warn that biprobit can be biased if the error distribution is misspecified, Li, Poskitt and Zhao [56] suggest that the use of instrumental variables increases the robustness of estimates of the ATE in such cases.

We estimate recursive biprobit models as our preferred specification. Although it is possible to rely on the non-linear biprobit functional form for identification [57] our inclusion of the distance difference IV may reduce problems due to misspecification [56]. We also report estimates of treatment effects from biprobit with no instrument, 2SLS, and single equation linear, probit, and logit mortality models.

Our biprobit specification is

$$D_{ir}^{*}=\lambda_{0}+z_{ir}\lambda_{1}+x_{ir}^{\prime }\lambda_{2}+x_{r}^{\prime }\lambda_{3}+\upsilon_{ir},\;D_{irt}=1(D_{irt}^{*}>0)$$ (2)

$$M_{ir}^{*}=\beta_{0}+\beta_{1}D_{ir}+x_{ir}^{\prime }\beta_{2}+x_{r}^{\prime }\beta_{3}+\varepsilon_{ir},\;M_{irt}=1(M_{irt}^{*}>0)$$ (3)

*D*_*ir*_ is an indicator for individual *i* resident in district *r* having dual insurance (belonging to SIS and ESSALUD) for at least seven consecutive days between 1 January 2020 and 5 March 2020. *M*_*ir*_ is an indicator for the individual dying from COVID-19 between 6 March 2020 and 30 June 2021. *υ* and *ε* have a joint standard normal distribution with correlation *ρ*.

**X**_*ir*_ is vector of characteristics of individual *i*: gender and age (≤ 19, 20–39, 40–59, 60–79, ≥ 80). **X**_*r*_ is a vector of the characteristics of the district of residence of the individual: indicators for the 25 department in which the district is located, note that with 24.7M observations and at most 25 dummy variables for each district characteristic, the estimated coefficients on the characteristics dummies will not suffer from the incidental parameters problem [58]; the geography of the region (capital city, coast, jungle, mountain) in which the district is located; rurality (indicator based on the number of households in the district); and three district poverty categories. It also includes controls for local hospital facilities: indicators for quintiles of the average distance from the district centroid to the nearest SIS and ESSALUD provider; quintiles of the average structural quality of the nearest SIS and ESSALUD providers, for this we compute the average level by interpreting the numerical label (1 to 3) for the structural quality level category of each hospital as a real number. We also include the proportion of the district population recorded as infected with COVID-19 between 6 March 2020 and 30 June 2021 to control for local unobserved factors affecting the risk of COVID-19 infection and thus COVID-19 mortality risk.

We use Stata 17 and the user written *cmp* package [59] to estimate the average treatment effect (ATE) and the average effect of treatment on the treated (AETT) of having dual rather than single insurance on COVID-19 mortality risk.

There is disagreement about whether and how standard errors should be clustered [60, 61]. Abadie, Athey [60] argue that clustering is not necessary when the estimation sample is not a draw from a larger population. MacKinnon, Nielsen and Webb [61] suggest that one should think of observations as draws from a meta sample of individuals and so clustering should be allowed for. Our sample is all individuals in SIS, ESSALUD or both at the beginning of the pandemic. We use individual level measures of outcome and treatment and control for individual characteristics such as age and gender. However, some of our explanatories are measured at district level and the instrument for dual insurance is based on the distance from the district centroid for the 38% of our sample for whom we do not have an exact address. We therefore use the more conservative approach of clustering robust standard errors at district level.

Our sample is very large, creating the risk that applying conventional levels of statistical significance will result in rejection of the null hypothesis of no effect of dual insurance even if the estimated effect is tiny and thus of little policy relevance [62, 63]. As we will see, the estimated ATE of dual insurance is large relative to the sample mean COVID-19 mortality risk as well as being very precisely estimated. We also find that the ATE is highly statistically significant even after adjusting *t* statistics upward to allow for sample size as suggested by Giles [64].

## 4 Results

### 4.1 Summary statistics

**Fig 2:**
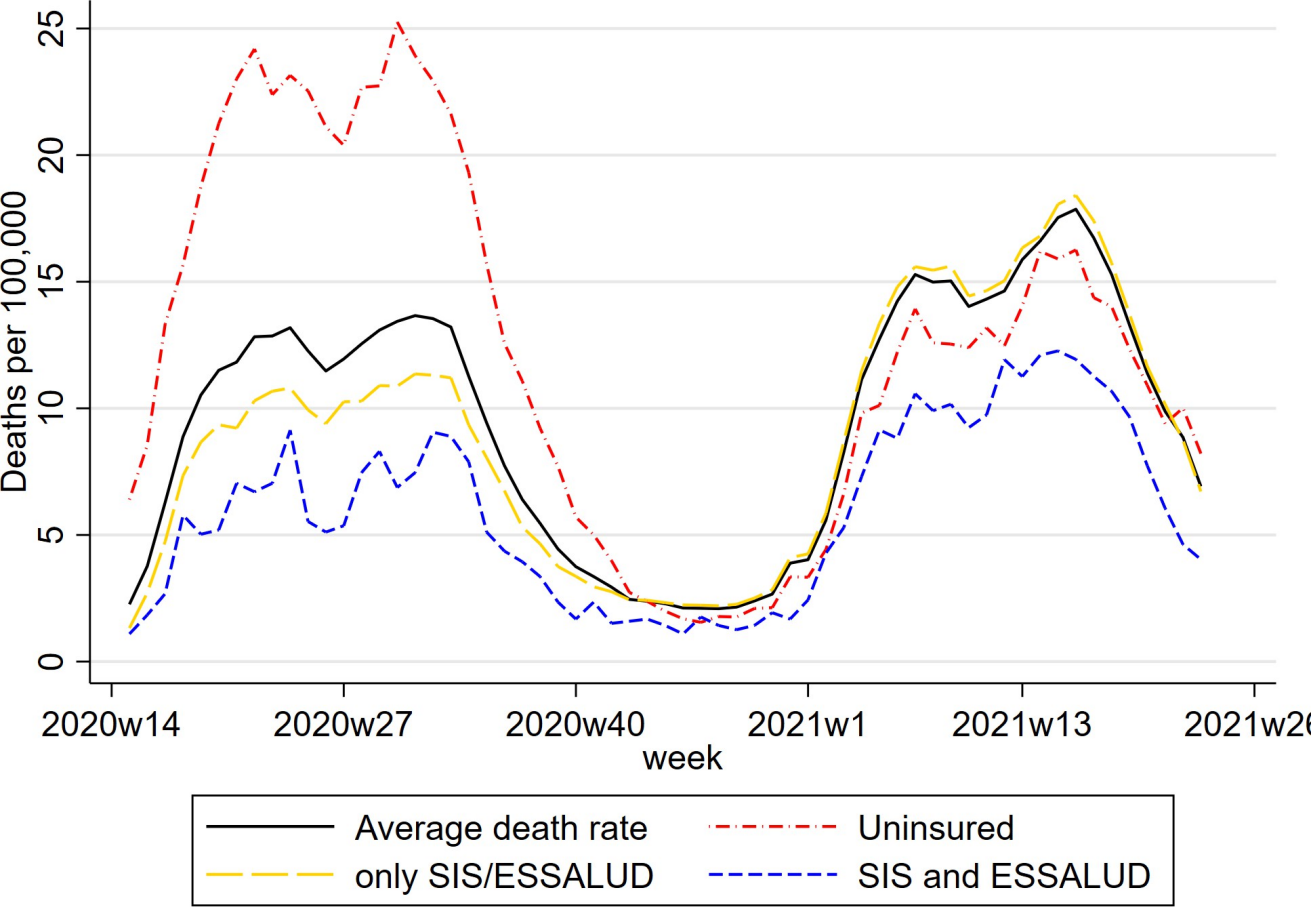
COVID-19 mortality rates by insurance status. Note: Insurance status at 31^st^ December 2019, mortality by COVID-19 followed up 30^th^ June 2020. Daily information averaged by week.

**Fig 2** plots COVID-19 mortality rate for groups of individuals defined by insurance status at date of death: insured with SIS or ESSALUD but not both, insured with both SIS and ESALUD, uninsured (no insurance of any kind: SIS, ESSALUD, private, police, or armed services) at the date of death. The COVID-19 mortality rate for the uninsured was much higher than for those with insurance during the first wave of the pandemic, despite membership of SIS being extended to the uninsured when they were diagnosed with COVID-19. During the second wave mortality amongst the uninsured was slightly below average, possibly because of mortality displacement, with the more susceptible being most likely to die in the first pandemic wave. Fig 2 also shows the COVID-19 mortality rate for individuals who were uninsured (not covered by SIS, ESSALUD, police, military, or private insurance). At the start of the pandemic SIS was extended to the uninsured when they were diagnosed with COVID-19 and on 28 July 2021, the bicentenary of the Peruvian republic, membership of SIS was extended to all uninsured individuals [34].

Our research question is whether having dual insurance (being in both SIS and ESSALUD) and thus having access to providers in both networks reduced COVID-19 mortality risk compared to being in only one the sub-systems and being able to access providers in one of the networks. In Fig 2 the mortality rate for those with dual insurance (blue line) is consistently below that of those with single insurance (yellow line). Fig 2 suggests that dual insurance is protective against COVID-19 but it is possible that the lower mortality risk of those with dual insurance is due to them being more likely to have characteristics other than insurance status which protect against COVID-19 mortality.

**Table 1** has summary statistics for our estimation sample of 24,739,933 individuals who were in SIS, ESSALUD, or both for at least seven consecutive days in 2019 and at least seven consecutive days between 1 January 2020 and 5 March 2020, with COVID-19 mortality recorded up to 30 June 2021. We define insurance status by whether the individual had dual insurance for at least seven consecutive days between 1 January and 5 March 2020. 0.55% of the sample (133,128 individuals) died from COVID-19 between 6 March 2020 and 30 June 2021. 5.8% of the sample had dual insurance and they had a lower mortality rate (0.37%) than those in only one of the insurance sub-systems (0.55%). Males had a much higher mortality rate than females (0.71% vs 0.38%) and were slightly more likely to have dual insurance than females (5.97% vs 5.70%). Dual insurance was associated with a bigger reduction in mortality risk for men than for women (0.26% vs 0.11%). Mortality risk was greater (0.87%) for those initially in ESSALUD than for those initially in SIS (0.37%), possibly because those in ESSALUD are older and located mainly in big cities where the pandemic spread more rapidly.

**Table 1:** Summary statistics: patient characteristics, dual insurance, COVID-19 mortality.

		Proportion of sample	Proportion with dual insurance	Mortality rate	Mortality rate by insurance status		Difference in mortality rate by insurance status
					Single insurance	Dual insurance	
*Full sample*							
All			0.0583	0.0054	0.0055	0.0037	0.00180***
Gender	Female	0.512	0.0570	0.0038	0.0038	0.0027	0.00111***
	Male	0.488	0.0597	0.0071	0.0072	0.0047	0.00259***
Age	0–19	0.335	0.0619	0.0001	0.0001	0.0001	-0.00001
	20–39	0.296	0.0702	0.0007	0.0007	0.0008	-0.00013***
	40–59	0.229	0.0559	0.0054	0.0054	0.0059	-0.00055***
	60–79	0.113	0.0331	0.0238	0.0236	0.0290	-0.00545***
	> 79	0.026	0.0088	0.0463	0.0463	0.0522	-0.00589*
ESSALUD		0.337	0.1036	0.0087	0.0093	0.0044	0.00487***
SIS		0.663	0.0354	0.0037	0.0037	0.0026	0.00107***
Region or residence	Lima	0.312	0.0802	0.0080	0.0083	0.0043	0.00405***
	Coast	0.249	0.0643	0.0061	0.0062	0.0040	0.00226***
	Jungle	0.146	0.0354	0.0028	0.0028	0.0025	0.00030*
	Mountains	0.293	0.0414	0.0033	0.0033	0.0026	0.00075***
Rurality of district of residence	Urban area	0.790	0.0661	0.0064	0.0066	0.0039	0.00265***
	Rural area	0.210	0.0290	0.0015	0.0015	0.0015	0.00002
Deprivation (more to least)	Group 1	0.366	0.0363	0.0022	0.0022	0.0020	0.00030***
	Group 2	0.426	0.0656	0.0064	0.0066	0.0038	0.00279***
	Group 3	0.208	0.0823	0.0088	0.0092	0.0048	0.00434***
Observations		24,739,933	1,443,205	133,128			

Note. Individuals alive at 5 March 2020 and with at least consecutive 7 days in ESSALUD or SIS between 1 January 2020 and 5 March 2020. Mortality followed from 6 March 2020 to 30 June 2021. ESSALUD (SIS): only have ESSALUD (SIS) or have dual insurance with ESSALUD (SIS) as first insurance. *, **, ***: p < 0.05, < 0.01, <0.001.

Mortality risk increased with age whilst dual insurance decreased. The age specific difference in mortality risk provides a nice example of Simpson’s paradox [65] in that, although mortality risk was smaller overall for those with dual insurance, mortality risk was higher in *every* age group for those with dual insurance.

Geography is strongly associated with dual insurance and with mortality and individuals in districts with higher dual insurance (such as the those in the capital city or an urban area) also have higher mortality. Both dual insurance and mortality rates are *inversely* related to deprivation, with those in the least deprived districts (Group 3) are more than twice as likely to have dual insurance as those in the most deprived districts (Group 1) and nearly four times as likely to die from COVID-19. This startling reversal of the usual relationship between deprivation and health is likely to be due to the lower risk of COVID-19 infection in rural areas with lower population density and greater deprivation.

**Table 2** compares providers available in the SIS and ESSALUD insurance sub-systems. SIS has more hospitals than ESSALUD, of all levels of structural quality, with 165 in total versus 71. Level 1 hospitals have 24-hour emergency services and operating theatres. Level 2 hospitals additionally have general intensive care units, and level 3 hospitals additionally have specialist intensive care units and research capacity. ESSALUD has fewer general and intensive care beds.

**Table 2:** Summary statistics: SIS and ESSALUD provider networks.

	ESSALUD	SIS	SIS and ESSALUD
Level 1	39	96	135
Level 2	21	46	67
Level 3	11	23	34
Total	71	165	236
Insureds per provider	158,286	119,886	131,439
Total hospital beds	13,670	16,689	30,359
Total ICU beds	171	238	409
Mean distance to level 1 providers	36.58	35.9	25.18
Mean distance to level 2 providers	76.74	45.49	34.23
Mean distance to level 3 providers	63.92	115.31	89.13

Note. Level 1 hospitals: have 24-hour emergency service and operating theatre; Level 2 as level 1 plus general Intensive Care Unit; Level 3 as level 2 plus Specialist Intensive Care Unit and research capacity. Insureds per SIS (ESSALUD) provider: individuals insured with SIS (ESSALUD) or both at 31 December 2019/total number of SIS (ESSALUD) providers. Mean distance: mean straight line distance (kms) from district centroid weighted by individuals in the district who were in SIS, ESSALUD or both at 31 December 2019. Number of hospital and ICU beds are at 30 April 2020.

ESSALUD has fewer insureds who are more geographically concentrated because they are more likely to live in large urban areas where formal employment is concentrated. Level 3 hospitals also tend to be located in these areas. Hence, although ESSALUD has fewer hospitals per insured, the average distance of ESSALUD insureds to level 3 ESSALUD hospitals is less than the average distance of SIS insureds to level 3 SIS hospitals. Level 1 providers in both networks are more dispersed and so average distances to them are similar for SIS and ESSALUD insureds. Distances to level 2 providers in their network are much greater for those in ESSALUD.

**Table 3** has summary statistics for the distance difference IV we use to predict whether an individual has dual insurance. The distance IV measures the *reduction* in distance to the nearest provider an individual initially insured with one sub-system would have if they were also a member of the other sub-system. We expect that individuals in only one sub-system are more likely to have a smaller improvement in access from dual insurance than those with dual insurance. This is so, on average, for individuals in ESSALUD. Those who have dual insurance and were initially in ESSALUD have a larger distance reduction (7.096km) from belonging to both sub-systems than those who are only in ESSALUD (4.563km). Because there are more SIS providers than ESSALUD providers the average distance to the nearest SIS provider is *smaller* than the distance to the nearest ESSALUD provider. However, it still the case that individuals with dual insurance who were initially in SIS but joined ESSALUD as well have a greater distance reduction from joining ESSALUD as well as SIS than the potential reduction for those who remain in SIS: −14.043 > −17.183. This suggests that for those initially in ESSALUD there is, on average, a bigger distance reduction from dual insurance than for those initially in SIS. However, these are averages, so that some individuals with dual insurance and initially in SIS will have a positive reduction if they could also access providers in ESSALUD.

**Table 3:** Summary statistics: Distance difference instrumental variable.

	Estimation sample	With only SIS	With dual insurance, initially in SIS	With only ESSALUD	With dual insurance, initially in ESSALUD
Observations	24,739,933	15,829,375	580,188	7,467,353	863,017
Proportion		0.640	0.023	0.302	0.035
*Distance difference IV (km)*					
Mean	-9.70	-17.18	-14.04	4.56	7.10
SD	36.49	37.53	33.44	30.08	26.07
Min	-208.22	-208.22	-208.22	-208.22	-80.65
Max	208.22	208.22	80.65	208.22	208.22
*Binary distance difference IV*					
Mean	0.384	0.274	0.283	0.594	0.655

Note. The distance difference IV is $\left(2S_{irt}^{first}-1)(d_{ir}^{SIS}-d_{ir}^{ESS}\right)$ where $S_{irt}^{first}$ is an indicator for a patient in the estimation sample initially being only in SIS and $d_{ir}^{SIS},d_{ir}^{ESS}$ are distances from the district centroid to the nearest SIS (ESSALUD) provider of any level. The binary version is $1(2S_{irt}^{first}-1)(d_{ir}^{SIS}-d_{ir}^{ESS})$. The statistics for IVs are weighted by the number of the relevant type of patients within the district who are in the estimation sample.

### 4.2 Model results

**Table 4** compares the estimates of the effect of dual insurance on COVID-19 mortality from four models which differ in whether they attempt to allow for endogeneity of dual insurance, for the binary nature of dual insurance and mortality, and in assumptions about functional form.

**Table 4:** Average treatment effect of dual insurance on COVID-19 mortality risk.

	(1)	(2)	(3)	(4)
	LPM	Probit	Biprobit	Biprobit
Instrument	No IV	No IV	No IV	Distance difference
ATE	-0.00044***	-0.00002	-0.00445***	-0.00227***
	(0.0001)	(0.0001)	(0.0003)	(0.0005)

Note. 24,739,933 observations in all models. Dual insurance: membership of SIS and ESSALUD for at least 7 days between 1 January 2020 and 5 March 2020. All models for mortality risk and dual insurance contain the full set of explanatory variables. F stat on IV equal to 257. Robust standard errors clustered at district level. *, **, ***: p < 0.05, < 0.01, <0.001.

The single equation linear probability and probit mortality models reported in columns (1) and (2) do not take account of the potential endogeneity of dual insurance. The single equation linear probability model (LPM) (column (1)) suggests that having dual insurance reduces mortality risk by 0.044%, compared to the sample mortality risk of 0.538%. Although the average estimated mortality risk is very similar to the sample mean risk, the LPM produces *negative* estimates of mortality risk for over 31% of the sample (see Table A3 in S2 Appendix).

The probit single equation mortality models cannot produce impossible estimates of individual mortality risk but yield very small (−0.002%) and statistically insignificant estimates of the average effect of dual insurance on mortality risk.

Columns (3) and (4) report the ATEs from biprobit models that allow for the potential endogeneity of dual insurance by using the distance difference IV in the first stage model for dual insurance. The IV is a strong predictor of dual insurance (F statistic 258), comfortably above the conventional threshold of 10 to avoiding the weak instrument problem in 2SLS [66]. Full results for these two models are in Table A4 in S2 Appendix. In column (3) the biprobit model has no IVs and identification is by functional form. In column (4) the biprobit models includes the distance difference IV. The ATEs are negative and precisely estimated in both biprobit models. Monfardini and Radice [67] suggests using a likelihood ratio test on the correlation of residuals as test of exogeneity in recursive biprobit models. A likelihood ratio test on the correlation of residuals [67] from column (3) biprobit model with no IV, suggests we reject the null hypothesis of exogeneity of dual insurance (χ^2^ = 1089.64, p < 0.01) and use the ATE of −0.227% from the biprobit model in column (4) with the distance IV as our preferred estimate. The *t* statistic for the ATE from the biprobit model with the differential distance IV (column (4)) is 5.13. The *t* statistic adjusted for sample size as suggested by Giles [64] is 4.13, implying that significance level of the ATE after allowing for our very large sample is 0.00018. We get similar patterns of results with a binary indicator version of the IV: $1(2S_{irt}^{first}-1)(d_{ir}^{SIS}-d_{ir}^{ESS})$ (Table A5 in S2 Appendix).

Our biprobit results contrast with what we can optain using linear 2SLS (See Table A6 in S2 Appendix). The estimated ATE of dual insurance using 2SLS is highly statistically significant but positive, suggesting that the average effect of dual insurance is to *increase* mortality risk by 3.432%. This seems highly implausible given that that the sample mortality risk is 0.538%. The average estimated mortality risk is, as with the linear model of column (1) in Table 4, very similar to sample mean risk. But the 2SLS mortality model produces nonsensical negative estimated mortality probabilities for 46% of the sample (see Table A3 in S2 Appendix).

### 4.3 Robustness to definition of dual insurance

In **Table 5** we investigate the sensitivity of the biprobit model results to the definition of dual insurance. Colaaumn (1) reproduces the results for having dual insurance for at least 7 days between 1 January 2020 and 5 March 2020. Columns (2) to (4) have results from successively looser definitions of dual insurance and any individual with dual insurance as defined in column (1) will also have dual insurance as defined in column (4). We suggested in Section 3.2 that as the definition of dual insurance became looser so that a larger proportion of the sample are recorded as having dual insurance, the estimated effect of dual insurance would become smaller, and this is what we observe in columns (2) to (4). For all of the pre-pandemic definitions of dual insurance in columns (1) to (4), the ATEs are all large relative to the mean mortality rate of 0.54%. With all four definitions, the ATE is larger in models without the IV for dual insurance, suggesting that there are unobserved characteristics which increase the probability that an individual has dual insurance and reduces their mortality risk.

**Table 5:** Sensitivity of average treatment effect to definition of dual insurance.

	Dual insurance pre-pandemic				Dual insurance during pandemic	
	(1)	(2)	(3)	(4)	(5)	(6)
	At least 7 consecutive days 01/01/20–05/03/20	All 7 days 25/12/19–31/12/19	At least 7 consecutive days 01/01/19–31/12/19	At least 7 consecutive days 01/01/19–05/03/20	At least 7 consecutive days 06/03/20–30/06/21	At least 1 day 06/03/20–30/06/21
ATE (Distance IV)	-0.00227***	-0.00315***	-0.00225***	-0.00175***	-0.00305***	-0.00278***
	(0.0005)	(0.0004)	(0.0004)	(0.0005)	(0.0003)	(0.0004)
ATE (no IV)	-0.00445***	-0.00480***	-0.00416***	-0.00403***	-0.00480***	-0.00473***
	(0.0003)	(0.0002)	(0.0003)	(0.0003)	(0.0003)	(0.0003)
Dual insurance (prop)	0.0583	0.0447	0.0763	0.0887	0.1069	0.1110

Note. 24,739,933 observations in all models. All models for mortality risk and dual insurance contain the full set of explanatory variables. Robust standard errors clustered at district level. *, **, ***: p < 0.05, < 0.01, <0.001.

The last two columns of Table 5 use definitions of dual insurance based on insurance status after the start of the pandemic. Both suggest a protective effect of dual insurance. However, it is possible that individuals may try to get dual insurance when they are infected (and so know that they are at greater risk of death from COVID-19), see Fig A5 in S2 Appendix which shows that dual insurance increased within 30 days of death from COVID-19, so that these results are not directly comparable with those based on pre-pandemic insurance.

### 4.4 Heterogeneity

**Table 6** reports the Average Treatment Effect (ATE) and the Average Treatment Effect on the Treated (ATET) for the full sample and for various patient subgroups. Overall COVID-19 mortality risk between 6 March 2020 and 30 June 2021 was 0.54%. We estimate that the average treatment effect (ATE) of dual insurance was to reduce mortality risk by 0.23% and the average effect on the treated (those with dual insurance) (AETT) was to reduce it by 0.17%. In Table 1 the mortality rates for those with dual insurance were higher than for those with single insurance in every age group, in apparent conflict with the lower mortality rate for those with dual insurance in the whole sample. The ATE and AETT subgroup results in Table 6 show that, after taking account of the endogeneity of dual insurance and the full set of covariates, mortality risk in every age group is *reduced* by dual insurance and there is a clear age gradient with dual insurance being more protective at higher ages.

**Table 6:** Treatment effects for subgroups.

		ATE	(SE)	AETT	(SE)
All		-0.00227***	(0.00045)	-0.00164***	(0.00047)
Gender	Male	-0.00300***	(0.00055)	-0.00215***	(0.00060)
	Female	-0.00154***	(0.00035)	-0.00111**	(0.00036)
Age	Age 0–19	-0.00005***	(0.00001)	-0.00007***	(0.00002)
	Age 20–39	-0.00037***	(0.00009)	-0.00043**	(0.00014)
	Age 40–59	-0.00277***	(0.00061)	-0.00321**	(0.00093)
	Age 60–79	-0.00956***	(0.00259)	-0.01117**	(0.00396)
	Age > 79	-0.01974***	(0.00531)	-0.02155**	(0.00728)
ESSALUD		-0.00855***	(0.00050)	-0.00536***	(0.00059)
SIS		-0.00331***	(0.00015)	-0.00201***	(0.00025)
Region	Lima (capital city)	-0.00284***	(0.0008)	-0.00183**	(0.0007)
	Coast	-0.00113	(0.0008)	-0.00070	(0.0006)
	Jungle	-0.00035	(0.0004)	-0.00027	(0.0004)
	Mountains	-0.00055	(0.0004)	-0.00039	(0.0004)
Rurality	Urban district	-0.00218**	(0.00064)	-0.00145**	(0.00056)
	Rural district	0.00016	(0.00031)	0.00009	(0.00021)
Deprivation of district	Group 1 (most)	-0.00008	(0.0004)	0.00005	(0.0003)
	Group 2	-0.00160*	(0.0007)	-0.00100	(0.0006)
	Group 3 (least)	-0.00288**	(0.0009)	-0.00189*	(0.0008)

Note. 24,739,933 observations in all models. Dual insurance: at least 7 days in 1 January 2020 to 5 March 2020. All models for mortality risk and dual insurance contain the full set of explanatory variables. Robust standard errors clustered at district level. *, **, ***: p value for difference < 0.05, < 0.01, <0.001.

Individuals initially in ESSALUD had a bigger reduction in mortality risk from dual insurance than those initially in SIS, perhaps reflecting the fact that getting dual insurance led to a greater increase in access to providers than for those initially in SIS. This is in line with the greater number of providers in the SIS network and shorter distances to them compared with providers in the ESSALUD network.

The effect of dual insurance depends on the type of geographical region, with the ATE and AETT being greatest for those living in capital city but with no effect for those in the other types of region. This may be because distances to all types of provider are greater, and transport facilities worse in the other types of region and so the *improvement* in access from belonging to both sub-systems has a smaller effect on mortality. Similarly, dual insurance reduces mortality in urban districts but not in rural districts. Dual insurance also reduced mortality risk for those in the least poor districts but had no effect for those in the poorest districts, possibly because poorer areas tend to be more likely to be rural.

## 5 Discussion

### 5.1 Comparison to findings from other research

Our research on the impact of dual insurance shows a reduction in mortality by 0.227 percentage points, compared to a sample mean of 0.538 percentage points. This translates to a 42% decrease in mortality (0.227/0.538), with a confidence interval ranging from 26% to 58%. Although this reduction might appear large, it is consistent with findings in existing literature.

Miller, Johnson, and Wherry [17] examined the effects of Medicaid expansion under the Affordable Care Act (ACA) in the United States, utilising extensive administrative datasets and a Difference-in-Difference methodology. Their findings suggest that the ACA expansion for individuals aged 55 to 64 reduced mortality by 34.9% to 63.2%. Additionally, when re-examining the Oregon Health Insurance Experiment [18] for the same age cohort, they observed a 71.7% reduction in mortality. They also reviewed several non-experimental studies on the ACA expansion’s impact, including younger age cohorts, referencing Sommers [68], Borgschulte and Vogler [69], and Chen [70]. Their review indicated that mortality reductions associated with ACA coverage expansion in non-experimental studies range from 9% to 65%.

Although our sample is not restricted to older age groups and encompasses the entire Peruvian population with SIS or ESSALUD at the pandemic’s outset, our analysis is comparable because COVID-19 mortality is predominantly concentrated among older age groups. As evidenced in Table 1, 95% of COVID-19 deaths in our analytical sample occurred in individuals aged 40 and above. Our results in Table 6 show that for individuals aged 40–59, there is a reduction in mortality by 0.277 percentage points, corresponding to a 51% decrease relative to the mean mortality for this age group. For those aged 60–79, a reduction by 0.956 percentage points implies a 40% decrease in mean mortality. For individuals over 79 years old, a reduction by 1.974 percentage points equates to a 43% decrease in baseline mortality. Hence, our findings are comparable to studies focusing on older age groups as discussed above, situating our results within the range found in previous literature.

In a context more closely related to insurance and COVID-19 mortality, Campbell et al. [71] posited that the fragmentation of the health system contributed to COVID-19 mortality in the United States. They estimated that universal health coverage could have reduced deaths by 26%, a figure that falls within the confidence intervals of the mortality reduction found in our study. Similarly, our findings suggest that one of the causes of higher mortality in Peru is the fragmentation of the health system.

Our findings diverge from those of Chakrabarti, Meyerson [20], who reported no significant differences in the logarithms of per capita COVID-19 cases and deaths across counties with Medicaid coverage in the United States. This discrepancy may be attributed to several factors. Firstly, unlike Miller, Johnson, and Wherry [17], who utilised individual-level administrative data, Chakrabarti’s study relied on county-level data. Despite encompassing over 3,000 counties with an average population of 100,000 per county [72], such aggregated data might obscure the potential effects of insurance on mortality rates. Secondly, the data used in their analysis was collected early in the pandemic, up to 18th September 2020, primarily capturing the first wave of COVID-19 mortality. This initial wave accounted for only approximately one-third of the deaths observed by the second wave and one-fifth by the third wave in the US [73]. Thus, re-estimating the model with more comprehensive data could potentially alter the reported outcomes. In contrast, our study includes COVID-19 deaths up to the second wave, which represent 90% of the COVID-19 deaths by the third wave in Peru [73], and utilises individual-level administrative data, enabling us to directly link insurance status with mortality at the individual level.

We acknowledge that comparing dual health insurance to single insurance differs from comparing single insurance to no insurance. However, due to the paucity of literature specifically addressing dual insurance, our most suitable comparison is with studies focusing on uninsured individuals. Another potential comparison involves examining studies that extend choice sets, which also provide evidence of reduced mortality. Dual insurance can effectively extend the choice set, offering access to more providers than single insurance.

In this context, Moscelli et al. [74] found that the reform undertaken to increase choice sets in the National Health Service in England in 2006 reduced mortality for hip fracture patients by 18%. Our higher results could be attributed to the fact that the increase in choice sets implemented in England applied to those already under single insurance, whereas in our case, choice sets for those with dual insurance increased access to two sub-systems. As shown in Table 2, on average, insureds in ESSALUD can more than double their choice set, while those in SIS can increase their choice by 40%. Thus, it is plausible to argue that dual insurance could have greater effects in enhancing choice sets and reducing mortality, making our results credible.

### 5.2 Effects of dual insurance in more deprived and rural areas

Table 6 indicates that dual insurance predominantly benefits urban districts, less deprived districts and Lima. As evidenced in Table 1, these regions exhibit the highest incidence of dual insurance, with a rate of 8% in Lima. In contrast, the Jungle region has a dual insurance rate of only 3.5%, and the Mountains, 4.1%. Overall, urban areas have a dual insurance coverage of 6.6%, more than double that of rural areas, which stands at 2.9%. Additionally, dual insurance is more prevalent in less deprived areas (group 3) at 8.2%, compared to 3.6% in the most deprived areas.

Table A7 in S2 Appendix provides comprehensive data on the average and maximum number of affiliations, reaffiliations, and disaffiliations among the 1,443,205 insured individuals in our sample with dual insurance (constituting 5.8% of the analytical sample). Disaffiliations are notably higher in more deprived areas, averaging 0.44, compared to 0.18 in less deprived areas. Similarly, rural areas experience a higher average number of disaffiliations (0.46) compared to urban areas (0.27). In Lima, the average number of disaffiliations is 0.19, approximately half of what is observed in other regions, such as the Jungle (0.40). Additionally, attempts at reaffiliation are observed up to 9 times in Lima, compared to up to 7 times in the Jungle.

This data suggests that Lima, urban areas, and less deprived areas benefit more significantly from dual insurance, as evidenced by higher coverage rates and lower disaffiliation rates. This may partly explain the observed effects of dual insurance in more advantaged areas. The policy implication is to promote dual insurance in rural and more deprived areas. It is also crucial to note the shortage of healthcare providers in rural regions, particularly in the Jungle, where accessing the nearest provider often involves travelling long distances. Therefore, the absence of notable results in rural areas should not be interpreted as an indication that dual insurance is ineffective; rather, it suggests the necessity for an expansion of healthcare services alongside the provision of dual insurance.

### 5.3 Increasing service interchange or granting dual insurance

It could be argued that Peru requires better coordination and service interchange between its health sub-systems rather than granting dual insurance to everyone or seeking unification of both health sub-systems. The concept of enhancing health system coordination or integration in Peru without unifying all public health services has been extensively addressed in Peruvian policy discussions, as evidenced by their official normative development. This effort can be traced back to the Acuerdo Nacional signed in 2002, where the main political parties and civil society representatives sought an integrated and coordinated health system [75].

In 2006, an official agreement was signed between ESSALUD and the Ministry of Health, which manages SIS, to interchange services [76]. Despite 27 specific agreements that followed, covering nearly all the Departments of Peru during the period 2012 to 2013, the evaluation commissioned by SIS for the period 2013 to 2016 indicated that the number of services exchanged decreased over the years and was almost insignificant in quantity. This decline was due to several burdens, including a lack of transparency in tariffs, delayed payments, differences in health package benefits, and changes in officials in charge of applying the agreements [77]. After this review was published, efforts to improve service interchange continued, with a focus on Cajamarca, a northern region of Peru, where the conditions for improving service interchange were promising. There is evidence that in 2021, the implementation of service interchange in Cajamarca enabled 99 SIS patients with cancer to be treated at ESSALUD. However, this interchange lasted only 18 months due to logistical problems with medicines [78].

The lack of success in articulating services between SIS and ESSALUD is further evidenced by the need for laws such as Legislative Decree 1159 issued in 2013 [79]. Article 4 of this decree mandates the obligatory interchange of health services between SIS and ESSALUD. The issuance of the law itself indicated that even after more than a decade of discussions since the Acuerdo Nacional, health services were still required to interchange and coordinate their services. To understand the importance of the Legislative Decree, it is important to note that it belongs to what is known as general law in Peru, only surpassed in authority by the Peruvian Constitution.

Despite this mandate, successful implementation was still lacking. This is evidenced by further legislation: Legislative Decree 1302 in 2016 [80], which aimed to optimise the exchange of health benefits in the public sector, and Legislative Decree 1466 in April 2020 [81], during the COVID-19 pandemic, which approved provisions to strengthen and facilitate the implementation of health benefits exchange in the national health system. All three laws mandate that service interchange is obligatory. However, all three laws also stipulate that the interchange of services is conditional on the sub-system’s capacity to offer necessary resolution capacity. Consequently, any provider can deny service interchange by claiming a lack of capacity due to their own insureds.

Patients seeking to access health services do not wait for the full implementation of service interchange, particularly ESSALUD insureds who can access Ministry of Health and regional government providers at a subsidised price. Table A8 in S2 Appendix shows that about half of ESSALUD insureds had at least one outpatient appointment with Ministry of Health providers during the period 2016 to 2019. Although it is less likely that ESSALUD patients would seek inpatient services such as surgeries, intensive care units, and hospitalisation due to higher costs than outpatient services, this indicates, at least to a certain extent, that while the disaffiliation of dual insurance seeks to prevent full cross-subsidisation from the Ministry of Health to ESSALUD, this cannot stop ESSALUD insureds from accessing Ministry of Health providers for outpatient services financed by general taxes, paying a partially subsidised tariff.

Practical and persistent articulation problems make the only straightforward option to efficiently access a wider network of providers to be enrolled simultaneously in SIS and ESSALUD or to unify SIS and ESSALUD into a single system. This research shows that about half a million individuals seek to gain access to dual insurance at any given time despite several efforts at disaffiliation (Fig 1). Furthermore, it shows that having dual insurance has health benefits, such as reducing mortality, which has been overlooked in the efforts at disaffiliation.

### 5.4 Relevance to other Countries and Health Conditions

The relevance of our results for other countries depends on the extent to which they have insurance sub-systems which restrict access to their provider networks. For example, in the US health insurers cannot refuse to pay for out of network emergency care, and major health insurance companies joined with Medicare and Medicaid and agreed to waive all copayments for treatment of COVID-19 [82]. But other countries in Latin America such as Brazil, Colombia and Mexico have had high COVID-19 fatality rates [1] and fragmented insurance sub-systems with restricted provider networks [83] and so might have benefited from removing restrictions on access.

Being in both SIS and ESSALUD reduced COVID-19 mortality because it gives access to the hospitals in the networks of both sub-systems. Time matters in the treatment of COVID-19 since a shorter time from onset to treatment reduces the probability of complications [84]. Rapid access to care is important not only in COVID-19 but also for outcomes in other conditions such as cancer [85], AMI [86], stroke [87], and serious mental illness [88]. Giving insureds who are only in one of the insurance sub-systems access to the provider networks in both sub-systems may therefore also have health benefits in the post-COVID-19 pandemic period.

### 5.5 Limitations

The main limitation of our analysis is the measure of dual insurance. We suggest that having dual insurance when infected with COVID-19 can reduce the risk of death from COVID-19 because having dual insurance gives access to the larger set of providers available in both networks. But we do not observe whether an individual who did not die from COVID-19 (99.46% of the sample) was infected with a COVID-19 infection and for the 0.54% who died from COVID-19 we do not observe when they were infected. Moreover, whether individuals have dual insurance can change over time as they claim and reclaim dual insurance status as their circumstances change and as the rules defining eligibility change. We argue that whether an individual had dual insurance for at least 7 days in the two months (1 January to 5 March 2020) before the outbreak of the pandemic in Peru is a good predictor of their ability to claim dual insurance when it is likely to be highly beneficial: when they are infected with COVID-19. Some individuals we define as dual insured did not have dual insurance when infected and some we define as not dual insured did have it when infected. These classification errors will tend to *underestimate* the true effect of having dual insurance when infected as they will increase the observed mortality rate amongst those we classify as dual insured and reduce it amongst those we classify as uninsured. Thus we argue that our finding that having dual insurance in the pre-pandemic period is associated with lower COVID-19 mortality risk is evidence for a genuine effect of having dual insurance when infected.

The probability of death from COVID-19 (mortality risk) is the probability of infection with COVID-19 multiplied by the probability of death from COVID-19 when infected (case fatality risk). With our data we cannot examine the separate effects of dual insurance on these two probabilities, only its effect on their product. Access to two sets of providers rather than one seems more likely to affect the COVID-19 case fatality risk rather than the risk of COVID-19 infection. However, mortality is not the only possible policy relevant outcome: a non-fatal infection with COVID-19 can increases the risk of worse health in the long term and increase health service costs.

## 6 Conclusion

Our study utilises the largest available administrative dataset, encompassing 24.7 million individual insurance records, which represents approximately 75% of the Peruvian population. We examined the impact of dual insurance on COVID-19 mortality in Peru, a country with the highest COVID-19 mortality rate globally. Employing a distance-based instrumental variable, we found that dual insurance—being simultaneously covered by SIS and ESSALUD, the two largest public health insurance providers—was associated with a 42% reduction in mortality, with a confidence interval ranging from 26% to 58%. This result is consistent with existing literature on the relationship between insurance coverage and mortality, indicating that universal dual insurance coverage could have potentially prevented between 34,894 and 78,069 COVID-19 deaths.

Our findings highlight that the fragmentation of Peru’s health insurance system significantly exacerbated the high COVID-19 mortality rate. Addressing this fragmentation—potentially through the expansion of dual insurance or the unification of the health system—could be instrumental in preparing for future pandemics and in improving healthcare delivery across various health conditions.

This suggests that policymakers should allow dual insurance for those insureds who potentially qualify for both SIS and ESSALUD under the current rules, and that consideration should be given to an experimental evaluation of removing the restriction that insureds can only access providers in their network in order to quantify the system wide health effects and possible costs.

## Supporting information

S1 Appendix Mortality data. (DOCX)

S2 Appendix Additional tables and figures. S2 Table A1. Case fatality rate by Covid-19 infection by insurance type from 6th March 2020 to 12th November 2020, S2 Table A2. Data cleaning, S2 Table A3. Predictions from linear models, S2 Table A4. Full results for biprobit models, S2 Table A5. Biprobit models with binary IV, S2 Table A6. 2SLS and biprobit models, S2 Table A7. Number of Affiliations and Disaffiliations of Dual Insurance Per Insured Before the Pandemic, S2 Table A8. Cross subsidy of ESSALUD insureds by the Ministry of Health’s providers in outpatient appointments, S2 Fig A1: The Peruvian health system, S2 Fig A2: Changes in dual insurance 1 January 2018–30 June 2021, S2 Fig A3: Cumulative affiliations and disaffiliations before and after start of pandemic, S2 Fig A4: Daily number of insureds by SIS, ESSALUD and dual insurance status, 1st January 2010–30 June 2021, S2 Fig A5: Evolution of insurance status 100 days up to date of death.
(DOCX)
