# Supplementary material for: Health insurance system fragmentation and COVID-19 mortality: Evidence from Peru
Source: PLoS One. 2024 Aug 27;19(8):e0309531. doi: 10.1371/journal.pone.0309531 (PMC11349220; doi:10.1371/journal.pone.0309531)
Supplement: S1 Appendix — (DOCX) [file pone.0309531.s001.docx]

**S1 Appendix: Mortality data**

Individual level mortality data is from the NOTI-SINADEF^[[1]](#footnote-1)^ dataset. Criteria for defining deaths from COVID-19 were revised on 31 May 2021 and applied retrospectively to data from 1 March 2021 (Health Ministry of Peru, 2021). The seven criteria are applied hierarchically to classify a death as due to COVID-19:

- Virological criterion: death in a confirmed case of COVID-19 within 60 days after a molecular test (PCR) or reactive antigen for SARS-CoV-2.
- Serological criterion: death in a confirmed case of COVID-19 within 60 days after a positive IgM or IgM / IgG serological test for SARS-CoV-2.
- Radiological criteria: death in a probable case of COVID-19 that presents a radiological, tomographic or nuclear magnetic resonance image compatible with COVID-19 pneumonia.
- Epidemiological link criterion: death in a probable case of COVID-19 that presents an epidemiological link with a confirmed case of COVID-19.
- Epidemiological investigation criteria: death in a suspected case of COVID-19 that is verified by epidemiological investigation of the National Epidemiology Network.
- Clinical criterion: death in a suspected case of COVID-19 that presents a clinical picture compatible with the disease.
- SINADEF Criterion: death with death certificate in which the diagnosis of COVID-19 is presented as the cause of death.

1. SINADEF is the Sistema Nacional de Defunciones, which is an administrative national system in which death certificates are recorded. [↑](#footnote-ref-1)
