# Supplementary material for: Health insurance system fragmentation and COVID-19 mortality: Evidence from Peru
Source: PLoS One. 2024 Aug 27;19(8):e0309531. doi: 10.1371/journal.pone.0309531 (PMC11349220; doi:10.1371/journal.pone.0309531)
Supplement: S2 Appendix — S2 Table A1. Case fatality rate by Covid-19 infection by insurance type from 6th March 2020 to 12th November 2020, S2 Table A2. Data cleaning, S2 Table A3. Predictions from linear models, S2 Table A4. Full results for biprobit models, S2 Table A5. Biprobit models with binary IV, S2 Table A6. 2SLS and biprobit models, S2 Table A7. Number of Affiliations and Disaffiliations of Dual Insurance Per Insured Before the Pandemic, S2 Table A8. Cross subsidy of ESSALUD insureds by the Ministry of Health’s providers in outpatient appointments, S2 Fig A1: The Peruvian health system, S2 Fig A2: Changes in dual insurance 1 January 2018–30 June 2021, S2 Fig A3: Cumulative affiliations and disaffiliations before and after start of pandemic, S2 Fig A4: Daily number of insureds by SIS, ESSALUD and dual insurance status, 1st January 2010–30 June 2021, S2 Fig A5: Evolution of insurance status 100 days up to date of death. (DOCX) [file pone.0309531.s002.docx]

**S2 Appendix: Additional Tables and Figures**

**Table A1:** **Case fatality rate by Covid-19 infection by insurance type from 6th March 2020 to 12th November 2020**

| **Type of insurance** | **Number of Covid-19 cases** | **% deaths** |
| --- | --- | --- |
|  |  |  |
| SIS+ESSALUD | 27,525 | 0.9% |
| SIS | 305,507 | 2.6% |
| ESSALUD | 373,426 | 2.2% |
| Police and military | 38,597 | 2.1% |
| Private | 11,942 | 2.7% |
| Uninsured | 78,971 | 29.0% |
| Total | 835,968 | 2.7% |

Source: RAUS SUSALUD for insurance and SISCOVID database for infection and deaths by Covid-19

**Table A2. Data cleaning**

| Number with insurance records at 05/03/2020 | |  |  | 32,278,940 |
| --- | --- | --- | --- | --- |
|  | Missing data on age, district of residence | | 61,653 |  |
|  | Not in SIS or ESSALUD* | | 7,477,354 |  |
| Total exclusions |  |  |  | 7,539,007 |
| Estimation sample |  |  |  | 24,739,933 |

* For at least one continuous week in 2019 and one continuous week from 01/01/2020 to 05/03/2020

**Table A3. Predictions from linear models**

|  | LPM | LPM | 2SLS (diff distance IV) | |
| --- | --- | --- | --- | --- |
| Predictions of | Dual insurance | Mortality | Dual insurance | Mortality |
| Mean | 0.05834 | 0.00538 | 0.05834 | 0.00538 |
| SD | 0.02863 | 0.01051 | 0.02863 | 0.01320 |
| Inter-quartile range | 0.04226 | 0.00649 | 0.04226 | 0.00822 |
| % < 0 | 2.91% | 30.57% | 2.91% | 46.31% |
| % > 1 | - | - | - | - |

**Table A4. Full results for biprobit models**

|  | Biprobit no IV | | Biprobit Distance IV | |
| --- | --- | --- | --- | --- |
|  | Dependent variable: dual insurance | Dependent variable: mortality | Dependent variable: dual insurance | Dependent variable: mortality |
| Dual insurance |  | -0.547*** |  | -0.215*** |
|  |  | (0.048) |  | (0.052) |
| Age 2 (20 - 39 years) | 0.051*** | 0.594*** | 0.054*** | 0.599*** |
|  | (0.006) | (0.015) | (0.006) | (0.015) |
| Age 3 (40 - 59 years) | -0.095*** | 1.229*** | -0.101*** | 1.254*** |
|  | (0.007) | (0.019) | (0.007) | (0.016) |
| Age 4 (60 - 79 years) | -0.362*** | 1.779*** | -0.367*** | 1.825*** |
|  | (0.006) | (0.023) | (0.006) | (0.019) |
| Age 5 (> 79 years) | -0.913*** | 2.070*** | -0.922*** | 2.135*** |
|  | (0.016) | (0.025) | (0.016) | (0.020) |
| Male | 0.029*** | 0.295*** | 0.028*** | 0.296*** |
|  | (0.003) | (0.004) | (0.003) | (0.004) |
| Geography |  |  |  |  |
| Lima Metropolitana | Ref. | Ref. | Ref. | Ref. |
|  |  |  |  |  |
| Coast | -0.061** | 0.009 | -0.063** | 0.013 |
|  | (0.023) | (0.017) | (0.022) | (0.017) |
| Jungle | -0.254*** | -0.117*** | -0.213*** | -0.106** |
|  | (0.038) | (0.033) | (0.038) | (0.034) |
| Mountains | -0.180*** | -0.123*** | -0.141*** | -0.116*** |
|  | (0.030) | (0.026) | (0.029) | (0.026) |
| Rural district | -0.110*** | -0.255*** | -0.099*** | -0.255*** |
|  | (0.014) | (0.016) | (0.014) | (0.016) |
| District deprivation |  |  |  |  |
| Group 1 (most deprived) | Ref. | Ref. | Ref. | Ref. |
|  |  |  |  |  |
| Group 2 | 0.097*** | 0.156*** | 0.082*** | 0.153*** |
|  | (0.016) | (0.019) | (0.016) | (0.019) |
| Group 3 (least deprived) | 0.259*** | 0.119*** | 0.246*** | 0.107** |
|  | (0.028) | (0.034) | (0.028) | (0.035) |
| Department 1 | -0.067 | -0.035 | -0.110** | -0.033 |
|  | (0.036) | (0.045) | (0.036) | (0.046) |
| Department 2 | 0.067** | 0.053** | 0.087*** | 0.052** |
|  | (0.025) | (0.019) | (0.024) | (0.019) |
| Department 3 | -0.038 | 0.011 | -0.090* | 0.013 |
|  | (0.038) | (0.036) | (0.039) | (0.036) |
| Department 4 | 0.039 | 0.067* | 0.009 | 0.067* |
|  | (0.028) | (0.032) | (0.027) | (0.032) |
| Department 5 | -0.081** | -0.018 | -0.108*** | -0.015 |
|  | (0.029) | (0.035) | (0.030) | (0.035) |
| Department 6 | -0.008 | -0.045 | -0.047 | -0.045 |
|  | (0.054) | (0.025) | (0.053) | (0.025) |
| Department 7 | 0.118*** | 0.088 | 0.115*** | 0.082 |
|  | (0.030) | (0.049) | (0.027) | (0.048) |
| Department 8 | 0.043 | 0.068** | -0.001 | 0.068** |
|  | (0.043) | (0.025) | (0.043) | (0.026) |
| Department 9 | 0.037 | 0.040 | -0.006 | 0.039 |
|  | (0.047) | (0.029) | (0.046) | (0.028) |
| Department 10 | -0.054 | 0.034 | -0.109*** | 0.037 |
|  | (0.030) | (0.024) | (0.032) | (0.024) |
| Department 11 | -0.031 | 0.106* | -0.037 | 0.109* |
|  | (0.033) | (0.046) | (0.032) | (0.046) |
| Department 12 | 0.020 | 0.142*** | -0.010 | 0.144*** |
|  | (0.029) | (0.026) | (0.028) | (0.026) |
| Department 13 | -0.005 | -0.040 | -0.005 | -0.041 |
|  | (0.023) | (0.022) | (0.022) | (0.022) |
| Department 14 | -0.098** | -0.025 | -0.103** | -0.020 |
|  | (0.037) | (0.021) | (0.035) | (0.021) |
| Department 15 (Lima) | Ref. | Ref. | Ref. | Ref. |
| Department 16 | 0.085 | 0.167*** | 0.033 | 0.166*** |
|  | (0.051) | (0.049) | (0.051) | (0.049) |
| Department 17 | -0.117** | 0.035 | -0.171*** | 0.044 |
|  | (0.045) | (0.052) | (0.044) | (0.052) |
| Department 18 | 0.110* | -0.092 | 0.101* | -0.100 |
|  | (0.046) | (0.061) | (0.043) | (0.062) |
| Department 19 | 0.199*** | 0.093** | 0.134** | 0.085* |
|  | (0.039) | (0.036) | (0.041) | (0.036) |
| Department 20 | 0.121*** | 0.070** | 0.115*** | 0.065* |
|  | (0.030) | (0.027) | (0.029) | (0.027) |
| Department 21 | -0.046 | 0.031 | -0.076* | 0.033 |
|  | (0.031) | (0.030) | (0.032) | (0.031) |
| Department 22 | -0.015 | -0.073 | -0.045 | -0.072 |
|  | (0.040) | (0.038) | (0.040) | (0.038) |
| Department 23 | -0.073 | -0.117** | -0.076 | -0.114** |
|  | (0.046) | (0.036) | (0.044) | (0.036) |
| Department 24 | -0.151*** | -0.042 | -0.155*** | -0.035 |
|  | (0.042) | (0.023) | (0.040) | (0.023) |
| Department 25 | -0.055 | 0.117** | -0.100* | 0.122** |
|  | (0.039) | (0.041) | (0.039) | (0.041) |
| Average structural quality nearest SIS and ESSALUD providers |  |  |  |  |
| Average level 1 | Ref. | Ref. | Ref. | Ref. |
| Average level 1.5 | 0.015 | 0.002 | 0.009 | 0.001 |
|  | (0.013) | (0.015) | (0.013) | (0.015) |
| Average level 2 | 0.042** | -0.015 | 0.037* | -0.018 |
|  | (0.015) | (0.016) | (0.015) | (0.016) |
| Average level 2.5 | 0.051* | -0.041 | 0.047* | -0.044* |
|  | (0.022) | (0.021) | (0.022) | (0.022) |
| Average level 3 | -0.004 | -0.101*** | -0.014 | -0.102*** |
|  | (0.021) | (0.024) | (0.021) | (0.024) |
| Average distance to providers in quintiles (lowest to highest) |  |  |  |  |
| q1 | Ref. | Ref. | Ref. | Ref. |
| q2 | 0.025 | 0.012 | 0.025 | 0.010 |
|  | (0.013) | (0.012) | (0.013) | (0.012) |
| q3 | -0.002 | 0.005 | -0.005 | 0.005 |
|  | (0.013) | (0.013) | (0.013) | (0.014) |
| q4 | -0.048** | -0.066*** | -0.038** | -0.065*** |
|  | (0.015) | (0.013) | (0.015) | (0.013) |
| q5 | -0.125*** | -0.110*** | -0.084*** | -0.105*** |
|  | (0.016) | (0.016) | (0.016) | (0.016) |
| District COVID-19 infection rate | 0.113 | 1.137*** | 0.109 | 1.142*** |
|  | (0.106) | (0.290) | (0.107) | (0.295) |
| Distance difference IV |  |  | 0.003*** |  |
|  |  |  | (0.0001) |  |
| Constant | 1.543*** | 3.994*** | 1.522*** | 4.065*** |
|  | (0.022) | (0.036) | (0.021) | (0.030) |
|  |  |  |  |  |
| Number of observations | 24,739,933 | 24,739,933 | 24,739,933 | 24,739,933 |
| Rho (correlation of residuals.) |  | 0.284*** |  | 0.103*** |
|  |  | (0.029) |  | (0.025) |

Note: Robust standard errors clustered at district level. *, **, ***: p value for difference < 0.05, < 0.01, <0.001.

**Table A5. Biprobit models with binary IV**

|  | Biprobit  Base case  (Continous IV) | Biprobit  Binary IV |
| --- | --- | --- |
| ATE | -0.00227*** | -0.00262*** |
|  | (0.0005) | (0.0005) |
| N | 24,739,933 | 24,739,933 |

Note: Robust standard errors clustered at district level. *, **, ***: p value for difference < 0.05, < 0.01, <0.001.

**Table A6.** **2SLS and biprobit models**

|  | Biprobit  (base case) | 2SLS |
| --- | --- | --- |
| ATE | -0.00227*** | 0.03505*** |
|  | (0.0005) | (0.0052) |
| N | 24,739,933 | 24,739,933 |

Note: Robust standard errors clustered at district level. *, **, ***: p value for difference < 0.05, < 0.01, <0.001.

**Table A7:** **Number of Affiliations and Disaffiliations of Dual Insurance Per Insured Before the Pandemic**

|  |  | Number of observations | Average Number of Affiliations and Reaffiliations | Average Number of Disaffiliations | Maximum Number of Affiliations and Reaffiliations | Maximum Number of Disaffiliations |  |
| --- | --- | --- | --- | --- | --- | --- | --- |
|  |  |  |  |  |  |  |  |
|  |  |  |  |  |  |  |  |
| Region or residence | Lima | 619,188 | 1.19 | 0.19 | 9 | 8 |  |
|  | Coast | 395,701 | 1.36 | 0.36 | 9 | 8 |  |
|  | Jungle | 128,026 | 1.40 | 0.40 | 7 | 6 |  |
|  | Mountains | 300,290 | 1.34 | 0.34 | 8 | 7 |  |
| Rurality of district of residence | Urban area | 1,292,322 | 1.27 | 0.27 | 9 | 8 |  |
|  | Rural area | 150,883 | 1.46 | 0.46 | 8 | 7 |  |
| Deprivation (more to least) | Group 1 | 328,597 | 1.44 | 0.44 | 8 | 7 |  |
|  | Group 2 | 691,794 | 1.28 | 0.28 | 8 | 7 |  |
|  | Group 3 | 422,814 | 1.18 | 0.18 | 9 | 8 |  |
| All insureds with dual insurance | | 1,443,205 | 1.29 | 0.29 | 9 | 8 |  |

Source: RAUS SUSALUD

**Table A8:** **Cross subsidy of ESSALUD insureds by the Ministry of Health’s providers in outpatient appointments**

|  | **2017** | **2018** | **2019** |
| --- | --- | --- | --- |
| Average number of ESSALUD insureds | 10,417,366 | 10,326,806 | 10,253,275 |
| ESSALUD insureds with an outpatient appointment in the Ministry of Health | 4,586,360 | 5,187,955 | 4,749,330 |
| % ESSALUD insureds in the Health Ministry | 44% | 50% | 46% |
| Number of outpatient consultancies from ESSALUD insured in the Health Ministry | 21,654,619 | 24,126,722 | 19,942,916 |

Source: RAUS SUSALUD for insurance, Health Information System - HIS for outpatient appointments.

**Figure A1: The Peruvian health system**

**
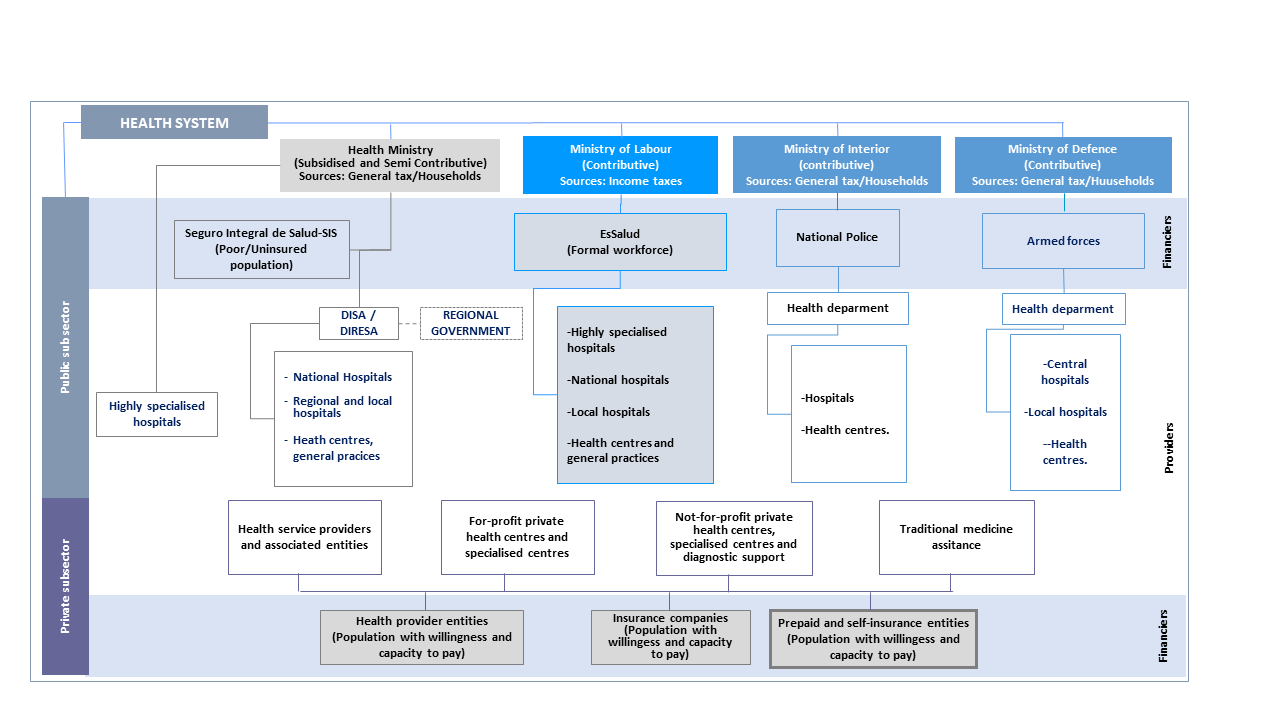
**

Note: Adapted from the presentation "Peru's Universal Health Coverage" by Virginia Baffigo de Pinillos, former Executive President of ESSALUD, at the Korea International Conference on Health Coverage in December 2014.

**Figure A2: Changes in dual insurance 1 January 2018-30 June 2021**

Source: RAUS-SUSALUD

Note: The figure shows the daily number of dual insurance changes (affiliations and disaffiliations). The vertical line indicates the start of the Covid-19 pandemic.

**Figure A3.** **Cumulative affiliations and disaffiliations before and after start of pandemic**

Source: RAUS-SUSALUD

Note: The figure shows the daily cumulative number of affiliations and disaffiliations to dual insurance (insureds who have SIS and ESSALUD simultaneously). The vertical line indicates the start of the COVID-19 pandemic.

**Figure A4: Daily number of insureds by SIS, ESSALUD and dual insurance status**

**1^st^ January 2010 - 30 June 2021**

Source: RAUS-SUSALUD

Note: The figure shows the daily number of insureds in SIS, ESSALUD and dual insurance (SIS and ESSALUD simultaneously). The vertical line indicates the start of the Covid-19 pandemic.

**Figure A5: Evolution of insurance status 100 days up to date of death**

Source: RAUS-SUSALUD for insurance, NOTI-SINADEF for Covid-19 mortality.

Note: The figure shows the affiliations and disaffiliations to ESSALUD, SIS, and dual insurance for people who died from COVID-19 up to 100 days before death. The vertical line indicates 30 days before death.
